# Creation of personalised rib prostheses using a statistical shape model and 3D printing: Case report

**DOI:** 10.3389/fsurg.2022.936638

**Published:** 2022-08-15

**Authors:** Antonia A. Pontiki, Savvas Lampridis, Sara De Angelis, Pablo Lamata, Richard Housden, Giulia Benedetti, Andrea Bille, Kawal Rhode

**Affiliations:** ^1^ Department of Surgical & Interventional Engineering, School of Biomedical Engineering and Imaging Sciences, King’s College London, London, United Kingdom; ^2^ Department of Thoracic Surgery, Guy’s and St Thomas’ NHS Foundation Trust, London, United Kingdom; ^3^ Department of Radiology, Guy’s and St Thomas’ NHS Foundation Trust, London, United Kingdom

**Keywords:** case report, chest wall, non-small cell lung cancer, prosthesis, statistical shape model, 3D printing

## Abstract

Management of chest wall defects after oncologic resection can be challenging, depending on the size and location of the defect, as well as the method of reconstruction. This report presents the first clinical case where patient-specific rib prostheses were created using a computer program and statistical shape model of human ribs. A 64-year-old male was diagnosed with non-small-cell lung cancer originating in the right upper lobe and invading the lateral aspect of the 3rd, 4th, and 5th ribs. Prior to surgical resection, a statistical shape model of human ribs was created and used to synthesise rib models in the software MATLAB (MathWorks, Natick, MA, USA). The patient's age, weight, height, and sex, as well as the number and side of the ribs of interest, were the inputs to the program. Based on these data, the program generated digital models of the right 3rd, 4th, and 5th ribs. These models were 3D printed, and a silicone mould was created from them. The patient subsequently underwent right upper lobectomy with *en bloc* resection of the involved chest wall. During the operation, the silicone mould was used to produce rigid prostheses consisting of methyl methacrylate and two layers of polypropylene mesh in a “sandwich” fashion. The prosthetic patch was then implanted to cover the chest wall defect. Thirty days after the surgery, the patient has returned to his pre-disease performance and physical activities. The statistical shape model and 3D printing is an optimised 3D modelling method that can provide clinicians with a time-efficient technique to create personalised rib prostheses, without any expertise or prior software knowledge.

## Introduction

As a result of advancements in healthcare technologies, particularly in imaging investigations and multimodality treatments, the number of patients with lung cancer undergoing surgery has increased. To provide adequate resection margins and improve patient prognosis, *en bloc* resection of lung cancers involving the chest wall is crucial (1). Lung resections that require chest wall excision account for approximately 5% of all lung tumours and present a three-times higher death rate than standard lung resections (2). This is mainly due to the impact of major chest wall resections on the mechanics of respiration. Therefore, reconstruction of the chest wall should restore the mechanics of the thorax, avoid paradoxical movement, and preserve the intrathoracic volume, with an acceptable cosmetic result (3).

Various techniques combining imaging and additive manufacturing have been previously used to create an improved shape for three-dimensional (3D) prostheses of thoracic structures. In recent years, additive manufacturing has been increasingly utilised in the medical field and has shown promising results in thoracic surgery, not only for the creation of personalised prostheses, but also for surgical planning, improving postoperative outcomes (4, 5). In our unit, the current method of reconstruction involves 3D printing and moulding of methyl methacrylate to produce a patient-specific prosthesis (6, 7). Image segmentation is used to reconstruct the patient's anatomy from preoperative computed tomography (CT) scans to generate personalised 3D rib prostheses that are manufactured using 3D printing and moulding.

The optimised, less labour-intensive method to produce a patient-specific, 3D digital rib model, described by Pontiki et al. (8), was used to create the prosthesis of a patient undergoing chest wall resection and reconstruction for non-small cell lung cancer. A custom software developed using MATLAB (MathWorks, Natick, MA, USA) and a statistical shape model were used to generate three rib models based on the patient's age, height, weight, and gender. We report the first clinical case where patient-specific rib prostheses were created using a computer program and statistical shape model of human ribs.

## Case description

**Figure 1:**
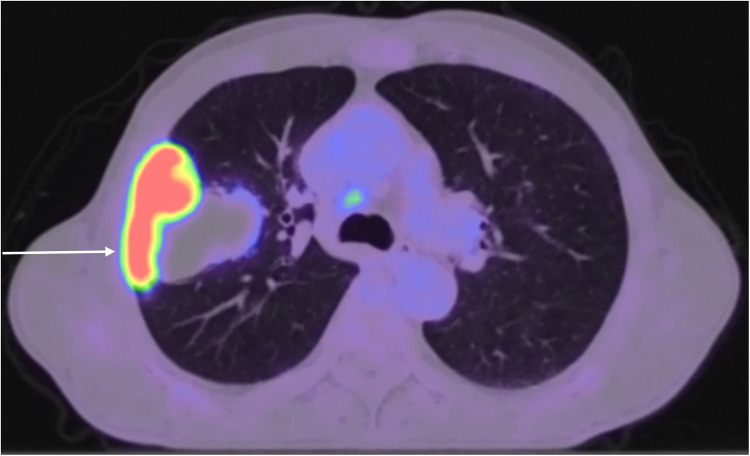
18F-fluorodeoxyglucose positron emission tomography integrated with CT showing heterogeneous radiotracer uptake within a pulmonary mass originating in the right upper lobe and infiltrating the chest wall, as well as low-grade metabolic activity in right lower paratracheal lymph nodes.

A 64-year-old man presented with productive cough, haemoptysis, and weight loss. He was a smoker who had accumulated an 80 pack-year history. His medical history also included chronic obstructive pulmonary disease and stage 3 chronic kidney disease. A contrast-enhanced thoracic computed tomography (CT) revealed a lobulated pulmonary mass within the right upper lobe measuring 68 × 60 × 79 mm and invading the lateral aspect of the 3rd, 4th, and 5th ribs, as well as marginally enlarged right paratracheal lymph nodes measuring up to 12 mm. A subsequent 18F-fluorodeoxyglucose positron emission tomography integrated with CT demonstrated heterogeneous radiotracer uptake within the mass, with a maximum standardised uptake value of 20.6, as well as low-grade metabolic activity in subcarinal and right lower paratracheal lymph nodes (Figure 1). Magnetic resonance imaging of the head showed no evidence of intracranial metastatic disease. A transthoracic core needle biopsy of the tumour under CT guidance indicated non-small cell lung cancer, not otherwise specified. Endobronchial ultrasound with transbronchial needle aspiration from lymph node stations 4L, 4R, and 7 did not reveal malignancy. The case was discussed at a multidisciplinary team meeting, with a consensus for multimodality treatment with upfront surgical resection. Preoperative pulmonary function tests showed forced expiratory volume in 1 s of 77% of predicted and diffusing capacity for carbon monoxide of 65% of predicted.

Surgery was performed under general anaesthesia with double-lumen tracheal intubation. The patient was placed in the lateral decubitus position and underwent a right posterolateral thoracotomy through the 6th intercostal space. The involved chest wall along with a 4-cm margin of normal tissue was removed *en bloc* with the right upper lobe. Systematic lymph node dissection was undertaken from stations 2R, 4R, 7, 9R, 10R, 11R, and 12R.

**Figure 2:**
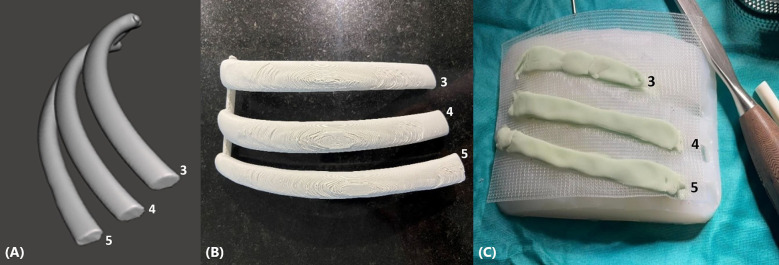
(**A**) 3D digital model of the right 3rd, 4th, and 5th rib sections to be resected and reconstructed. (**B**) 3D printed right 3rd, 4th, and 5th rib sections in polylactic acid, used to create the silicone mould. **(C)** Polypropylene mesh and methyl methacrylate were placed on a silicone mould, which was created from 3D printed rib segments, to create a personalised composite implant.

In this case report, the optimised 3D modelling method described by Pontiki et al. (8) was used to create 3D models of the 3rd, 4th, and 5th right ribs prior to the surgery. The software MATLAB was used, where a program was created to generate rib digital models within a few seconds, using a rib statistical shape model. The MATLAB code takes as inputs the patient's age (years), height (cm), weight (kg), and sex, as well as the number and side (left or right) of ribs. Based on a rib statistical shape model, the software generates a digital model for a corresponding rib mesh that would be representative of an “average” patient within this specified demographic. For our patient, the process was repeated three times to generate the three ribs of interest. Therefore, the inputs to the MATLAB code were 64, 177, 70.5, male, and 3, 4, or 5 for the first, second and third iteration, respectively. The three rib models were generated and saved as a mesh in a stereolithography (STL) file format. The digital models were processed using the software Meshmixer (Autodesk, San Rafael, CA, USA) to keep only the rib segments that were involved by the tumour, and hence they would be resected and reconstructed during surgery (Figure 2A). This method of creating the 3D meshes of the three ribs and processing the meshes lasted approximately 6 min. The slicing software Cura version 4.8 (Ultimaker, Utrecht, Netherlands) was then used to convert the rib meshes into G-code, a series of instructions that the 3D printer can process. The G-code was imported into the 3D printer (Ender-3, Creality, Shenzhen, China), which printed the ribs using polylactic acid filament (Polyplus, Polymaker, Shanghai, China) (Figure 2B). The printing of the ribs was completed in approximately 6 h.

**Figure 3:**
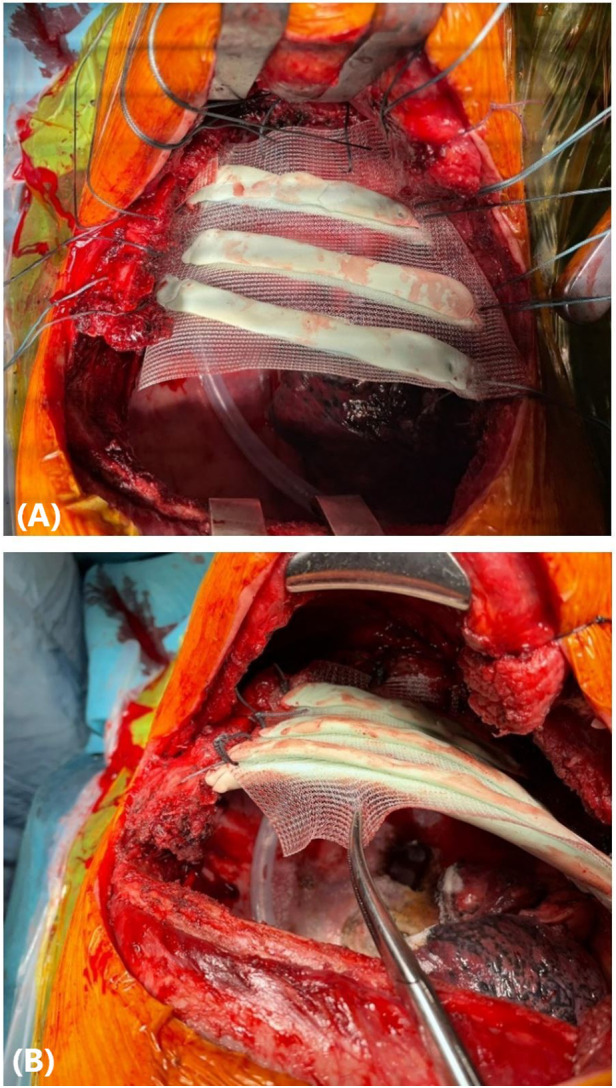
(**A**) A personalised prosthesis made of methyl methacrylate and polypropylene mesh in a “sandwich” fashion was implanted to reconstruct a chest wall defect involving the 3rd, 4th, and 5th ribs. (**B**) The prosthetic patch was secured to the chest wall with heavy gauge, nonabsorbable, polyfilament sutures.

The rib prostheses were manufactured using the silicone mould method described previously by Pontiki et al. (6, 7). The silicone mould was sterilised prior to the surgery and was used in the operating room under sterile conditions. Methyl methacrylate was mixed into a paste and applied to the silicone mould to create the prostheses of the resected segments of the 3rd, 4th, and 5th ribs. Two layers of polypropylene mesh (Prolene, Ethicon, Bridgewater, NJ, USA) were placed on the cement to create a “sandwich”. The meshes were tailored accordingly to leave 3 cm of prosthetic material for securing the patch to the chest wall (Figure 2C). Once the methyl methacrylate solidified after approximately 7 min, the prosthesis was removed from the mould. Suture holes were drilled in the edges of the prosthetic and corresponding autologous ribs. Heavy gauge, nonabsorbable, braided sutures (Ethibond, Ethicon, Somerville, NJ, USA) were placed through the holes and tied. The polypropylene meshes were secured on the surrounding intercostal muscles with Prolene sutures (Figure 3). A 28-Fr drain was inserted prior to closure. The latissimus dorsi and serratus anterior muscles were reattached to the prosthesis to increase stability, and the incision was closed in a standard fashion.

**Figure 4.**
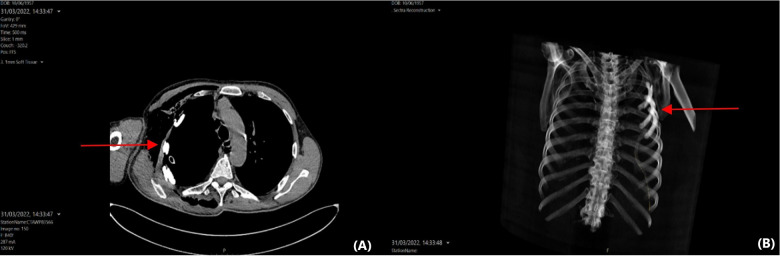
(**A**) Axial computed tomography of the thorax showing a personalised prosthesis contoured to the concavity of the chest wall and residing anatomically. (**B**) 3D reconstruction of the same computed tomography scan.

Postoperatively, the patient was transferred to the ward (level 1 of care). The only postoperative complication was a prolonged air leak due to the history of chronic obstructive pulmonary disease and multiple adhesions between the right lung and chest wall. The air leak was successfully treated with autologous blood pleurodesis. Thirty days after his surgery, the patient has remained free of other adverse effects. He has gradually returned to his pre-disease performance status, and he is satisfied with the functional and cosmetic result ((Figure 4). Histopathological analysis of the resected specimens revealed a large-cell carcinoma measuring 82 mm in maximal dimension, which was completely resected. The pathological classification of the cancer was pT4N0M0 (stage IIIA) according to the 8th edition of the international staging of thoracic malignancies.

## Discussion

In cases of locally advanced lung cancer involving the chest wall, surgical resection with adequate margins is considered the best treatment. Such operations may result in defects that require stabilization in order to restore the structural integrity of the chest wall and reduce the risk of postoperative complications, with an aesthetic reconstruction. Over the years, several methods have been used to achieve alloplastic reconstruction of rib defects with preservation of the original anatomical shape. As an example, Suzuki et al. (9) used a Steinmann pin and Penrose drain to form a neo-rib made by methyl methacrylate. Other authors have used automatic segmentation methods to construct the 3D shape of rib prostheses. Staal et al. (10) implemented a 5-step framework, in which voxels describing the target rib were detected and used to create primitives, which were further classified and grouped to fully segment the anatomy. Belal et al. (11), on the other hand, proposed a deep learning-based technique. A convolutional neural network (CNN) was used to identify a set of anatomical landmarks, which were then fed to another CNN that performed the final rib segmentation.

Our previously described technique of chest wall reconstruction involves the construction of the digital rib models by semi-automatically segmenting the patient's preoperative CT scan (6, 7). This method produced a customed rib prosthesis with successful clinical outcomes. However, it is labour-intensive and time-consuming. Indeed, semi-automatic image segmentation took 11.56 ± 1.60 min to reproduce the shape of a single rib. Similarly, the automatic rib segmentation method described by Staal et al. (10) took approximately 6.5 min, and the CNN-based method by Belal et al. (11) took 2 min per CT to generate automated segmentations. However, the method described in the present report takes 0.027 ± 0.009 min to generate the shape of a single rib. Specifically, the creation of the 3D meshes for the three rib segments took 5.081 ± 0.013 min to achieve the final digital model of the prosthesis, including the time to cut the segment of the rib that required resection and reconstruction. Moreover, segmentation methods require access to CT scans and good knowledge of the segmentation software, which is more complex and uses a multi-step process, compared to the software used in this case which involves inputting five values and running the program. Any member of the clinical care team with no specific expertise, could be quickly trained to use the software described in this method, in order to generate the rib models in less than a minute.

The method described here is characterised by some limitations. The most significant limitation is that the new statistical shape model method does not provide the patient with an identical replication of their original anatomy. The prosthesis is anatomically accurate for any patient with the characteristics used as input for the software, with average error of less than 2 mm (8). However, this patient's postoperative cosmetic result was satisfactory, with restoration of the natural chest contour. Moreover, this method requires access to and knowledge of operating a 3D printer, any member of staff though could easily be trained on this technology. This method introduces an extra cost of £184 to the standard technique, including the printer cost itself. The 3D printed models and silicone mould for each case adds on average £33 to the standard MMA technique, which is not significant in the context of a major resection surgery, or compared to customized titanium alternatives available on the market, costing $1,200 for 2 ribs (12). Finally, spirometry was not used to assess potential changes in lung function; in our practice, we only perform pulmonary function tests postoperatively if they are clinically indicated.

In conclusion, this case highlights the ease-of-use and efficiency of the proposed new method of the 3D prosthesis design. This technique further developed the previously used method of 3D printing and a silicone mould for chest wall reconstruction, providing the advantage of the software's time efficiency. It is completely reproducible since it can be used for any input of patient characteristics, does not require trained staff with knowledge of thoracic anatomy, and reduces the time of data acquisition and processing, achieving a satisfactory prosthesis, without significant loss of fidelity. The optimised technique could increase productivity due to its time-efficiency and could make this method more accessible in the clinical setting.

## Data Availability

The original contributions presented in the study are included in the article/Supplementary Material, further inquiries can be directed to the corresponding author/s.
